# Functional recovery in patients with schizophrenia: recommendations from a panel of experts

**DOI:** 10.1186/s12888-018-1755-2

**Published:** 2018-06-05

**Authors:** Guillermo Lahera, José L. Gálvez, Pedro Sánchez, Miguel Martínez-Roig, J. V. Pérez-Fuster, Paz García-Portilla, Berta Herrera, Miquel Roca

**Affiliations:** 1 Departamento de Medicina y Especialidades Médicas, Facultad de Medicina, Universidad de Alcalá, Plaza de San Diego, s/n, 28801 Alcalá de Henares, Madrid, Spain; 2 Centro de Investigación Biomédica en Red de Salud Mental (CIBERSAM), Madrid, Spain; 3 Unidad de Salud Mental Comunitaria del Hospital Universitario Virgen del Rocio, Av. Manuel Siurot, S/N, 41013 Sevilla, Spain; 4 Unidad de Psicosis Refractaria, Hospital Psiquiátrico de Álava-Osakidetza, C/ Álava, n°45, 01006 Vitoria – Gasteiz, Spain; 5 Facultad de Medicina, Universidad del País Vasco-Euskal Herriko Unibertsitatea, Leioa, Spain; 6 Hospital Royo Villanova, Avda. San Gregorio, s/n, 50015 Zaragoza, Spain; 7 Centro de Salud Mental Fuente San Luis, Hospital Universitario Doctor Peset, Av. de Gaspar Aguilar, 90, 46017 València, Spain; 8 Área de Psiquiatría, Facultad de Medicina, Universidad de Oviedo, Av. Julián Clavería, s/n, 33006 Oviedo, Asturias Spain; 9 Medical Affairs Department, Janssen-Cilag, Avenida Partenón, 16 1 (Campo de las Naciones), S. A, 28042 Madrid, Spain; 10 Institut Universitari d’Investigació en Ciencies de la Salut (IUNIS), RedIAPP, Hospital Juan March, Universidad de las Islas Baleares, Cra. de Valldemossa, km 7.5., Palma de Mallorca, (Illes Balears) Spain

**Keywords:** Schizophrenia, Functional recovery, Psychosocial therapy, Antipsychotic agents

## Abstract

**Background:**

The management of schizophrenia is evolving towards a more comprehensive model based on functional recovery. The concept of functional recovery goes beyond clinical remission and encompasses multiple aspects of the patient’s life, making it difficult to settle on a definition and to develop reliable assessment criteria. In this consensus process based on a panel of experts in schizophrenia, we aimed to provide useful insights on functional recovery and its involvement in clinical practice and clinical research.

**Methods:**

After a literature review of functional recovery in schizophrenia, a scientific committee of 8 members prepared a 75-item questionnaire, including 6 sections: (I) the concept of functional recovery (9 items), (II) assessment of functional recovery (23 items), (III) factors influencing functional recovery (16 items), (IV) psychosocial interventions and functional recovery (8 items), (V) pharmacological treatment and functional recovery (14 items), and (VI) the perspective of patients and their relatives on functional recovery (5 items). The questionnaire was sent to a panel of 53 experts, who rated each item on a 9-point Likert scale. Consensus was achieved in a 2-round Delphi dynamics, using the median (interquartile range) scores to consider consensus in either agreement (scores 7–9) or disagreement (scores 1–3). Items not achieving consensus in the first round were sent back to the experts for a second consideration.

**Results:**

After the two recursive rounds, consensus was achieved in 64 items (85.3%): 61 items (81.3%) in agreement and 3 (4.0%) in disagreement, all of them from section II (assessment of functional recovery). Items not reaching consensus were related to the concepts of functional recovery (1 item, 1.3%), functional assessment (5 items, 6.7%), factors influencing functional recovery (3 items, 4.0%), and psychosocial interventions (2 items, 5.6%).

**Conclusions:**

Despite the lack of a well-defined concept of functional recovery, we identified a trend towards a common archetype of the definition and factors associated with functional recovery, as well as its applicability in clinical practice and clinical research.

**Electronic supplementary material:**

The online version of this article (10.1186/s12888-018-1755-2) contains supplementary material, which is available to authorized users.

## Background

The management of schizophrenia has traditionally focused on the assessment of symptomatology and neurocognitive functioning [1]. However, there is increasing interest in developing more comprehensive models focusing on functional recovery [2–9]. Unlike clinical remission, which is well defined and can be measured, the concept of recovery encompasses multiple aspects of the patient’s life, making it difficult to settle on a definition and to develop reliable assessment criteria. Liberman and colleagues [10] proposed operational criteria for recovery from schizophrenia that included symptom remission, improved vocational functioning, independent living, and improved peer relationships. In contrast, Anthony [11] described functional recovery as a deeply personal, unique process of changing one’s attitudes, values, feelings, goals, skills, and/or roles, even with limitations caused by illness. On the other hand, some clinicians have warned that functional recovery can only be accepted when symptoms are mild and stable enough to not interfere with normal functioning in social activities and relationships [6, 12]. Regardless of the perspective of the various stakeholders, it is widely accepted that functional recovery is influenced by the severity of symptoms as well as by disease-related aspects such as neurocognitive performance [7, 13–15]. Additionally, social and family circumstances, opportunities, and lifetime events contribute to extending the list of environmental factors that may influence functional recovery beyond clinical manifestations of schizophrenia [7, 15, 16].

Patients with schizophrenia now have access to a wide variety of pharmacological agents and psychosocial therapies which may eventually meet the particular needs of each patient profile and, therefore, increase the chances of positive therapeutic outcomes [17]. The lack of standardized tools for the assessment of functional recovery prevents from drawing strong conclusions regarding the contribution of these interventions to functional recovery in patients with schizophrenia. Nevertheless, results of clinical studies – including randomized controlled trials – on various interventions for schizophrenia suggest that the achievement of functional recovery is possible in many cases [18–21].

Due to the heterogeneity of published information and the limited empirical evidence on functional recovery, this concept is not commonly considered an assessment criterion and/or a therapeutic goal in most clinical practice guidelines [1]. In a non-standardized way, most clinicians are familiar with the concept of functional recovery and consider it useful in their day-to-day practice [22]. Nevertheless, it is not clear whether clinicians have a common construct of functional recovery and to what extent these ideas meet the empirical evidence published in the literature. We present herein the results of a Delphi consensus process aimed to identify commonly accepted concepts regarding the definition and assessment of functional recovery, as well as the perceived impact of psychosocial and pharmacological interventions on its attainment.

## Methods

### Design of the consensus dynamics

The aim of this consensus was to apprise various aspects related to the assessment and functional intervention of patients with schizophrenia and provide clinicians and investigators with insights into functional recovery in patients with schizophrenia. The consensus process was approached through two recursive rounds of Delphi dynamics. The Delphi methodology is a structured, systematic, and interactive forecasting method based on the individual judgments of a panel of experts [23, 24]. The two Delphi rounds were held between May 5th 2016 and July 1st 2016. In each round, experts anonymously acceded to an online questionnaire and rated each item in the questionnaire on a 9-point Likert scale, where lower scores meant disagreement and higher scores meant agreement. For each item, consensus was considered when at least two-thirds of the experts scored either 1-to-3 (disagree) or 7-to-9 (agree) (Additional file 1: Table S1). Conversely, non-consensus was considered when the interquartile range (i.e. percentiles 25–75) was greater than 4 or when one-third of the experts or more scored either 1-to-3 or 7-to-9. In that case an indeterminate level was assigned to items not meeting the criteria for either consensus or non-consensus. Items not achieving consensus in the first round were sent back to the experts for a second assessment round.

Using bar graphs, a research assistance team, which did not interfere in the responses, assessed and presented the results from the first round to facilitate comments and clarifications from each participant. In the second round, the expert panelists contrasted their personal opinion with the result of the first round and, if necessary, reconsidered their initial opinion on those items in which consensus was not reached. The results of this second round were tabulated and presented descriptively using the median and interquartile range and the percentage of experts agreeing/disagreeing a particular statement.

At the end of the Delphi process, the scientific committee discussed the final results during a group session, held on October 15th, 2016. A manuscript was drafted with the conclusions drawn from the responses of the panel of experts and the literature review, which was revised and approved by all members of the scientific committee.

### Participants

The scientific committee consisted of eight psychiatrists from Spanish hospitals. Each member of the scientific committee proposed and recruited psychiatrists from Spanish hospitals, outpatient units, and academic settings to participate in the panel of experts. To be included in the panel, the expert must have at least 10 years of experience in a hospital setting. To prevent attrition bias between the two rounds, only experts who completed the entire Delphi process were to be listed in the acknowledgement section of the publication [25]. The research assistance team, led by BH, directed and oversaw the entire process and was responsible for the distribution and analysis of the questionnaires.

### The questionnaire

After a literature review of the empirical evidence and expert opinions on functional recovery in patients with schizophrenia, the members of the scientific committee discussed the unmet needs regarding the definition of functional recovery and its assessment, as well as the possible interventions to accomplish it. The items of interest were summarized in a questionnaire of 75 items, which were grouped into six sections: (I) the concept of functional recovery (9 items), (II) assessment of functional recovery (23 items), (III) factors influencing functional recovery (16 items), (IV) psychosocial interventions and functional recovery (8 items), (V) pharmacological treatment and functional recovery (14 items), and (VI) the perspective of patients and their relatives on functional recovery (5 items). The questionnaire was sent to the experts in the panel for their consideration.

## Results

### Consensus overview

**Fig. 1:**
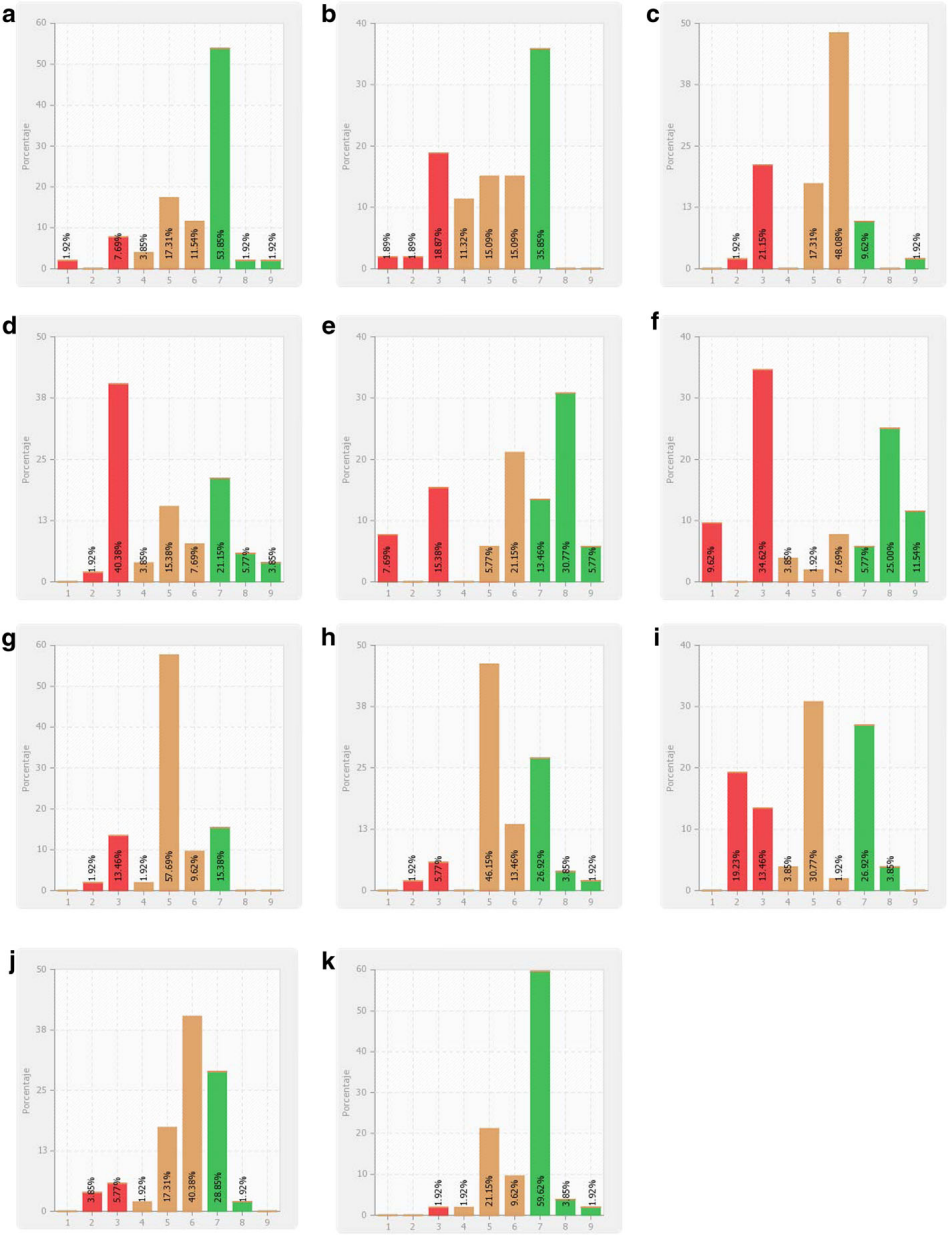
Results of the items not meeting the consensus criteria. **a** functional recovery is a well-established concept. **b** The functional assessment is commonly included among the objectives of clinical trials on schizophrenia. **c** the patient is the most reliable source of information for functional assessment. **d**: a proper functional assessment is not affordable in institutionalized patients. **e** I am familiar with the use of the Social and Occupational Functioning Assessment Scale (SOFAS) regarding the functional assessment of schizophrenic patients. **f** I am familiar with the use of the Health of the Nation Outcome Scales (HoNOS) regarding the functional assessment of schizophrenic patients. **g** living in an urban area is the environmental factor that has the greatest impact on functioning in patients with schizophrenia. **h** being an immigrant is the environmental factor that has the greatest impact on functioning in patients with schizophrenia. **i**: Public campaigns aimed at reducing stigma in patients with schizophrenia are effective. **j** cognitive-behavioral therapy is the most effective psychosocial intervention for functional recovery. **k** cognitive disorders should be the primary target of psychosocial interventions

Based on the criteria of the members of the scientific committee, 80 experts were invited to participate in the Delphi process. Of them, 53 responded the first round and 53 in the second one, indicating no drop out between the two recursive rounds of the Delphi process. In the first round, consensus was achieved in 41 items (54.7%), all of them in agreement. Of the 34 items addressed in the second round, 23 met the consensus criteria (20 items in agreement and 3 in disagreement), yielding a final consensus list of64 items (85.3%): 61 (81.3%) in agreement and 3 (4.0%) in disagreement. Table 1 shows the number and percentages of items in each section on which the experts reached agreement (i.e. consensus on the score range 7-to-9), disagreement (i.e. consensus on the score range 1-to-3), and lack of consensus. Of eleven items not meeting the consensus criteria, one resulted in non-consensus (i.e. more than one-third of experts scoring 1-to-3 and more than one-third scoring 7-to-9) and ten were indeterminate (i.e. not meeting criteria for either consensus or non-consensus) (Fig. 1). Most items not reaching consensus belonged to the functional assessment section (5 items, 6.7%), followed by factors influencing functional recovery (3 items, 4.0%), psychosocial interventions and functional recovery (2 items, 5.6%), and the concept of functional recovery (1 item, 1.3%). Additional file 1: Tables S2-S7 provide details on the median and interquartile range obtained on each item. The median score in items meeting the consensus criteria ranged from 6.06 to 8.31.

**Table 1:** Consensus rate in each section of the questionnaire

	No.	%
I. The concept of functional recovery (*n* = 9)		
Agreement	8	88.9%
Disagreement	0	–
No consensus	1	11.1%
II. Functional assessment (*n* = 22)		
Agreement	15	68.2%
Disagreement	3	13.6%
No consensus	5	22.7%
III. Factors influencing functional recovery (*n* = 16)		
Agreement	13	81.3%
Disagreement	0	–
No consensus	3	18.8%
IV. Psychosocial interventions and functional recovery (*n* = 8)		
Agreement	6	75.0%
Disagreement	0	–
No consensus	2	25.0%
V. Functioning and pharmacological treatment (*n* = 14)		
Agreement	14	100%
Disagreement	0	–
No consensus	0	–
VI. the perspective of patients and their relatives on functional recovery (*n* = 5)		
Agreement	5	100%
Disagreement	0	–
No consensus	0	–

### The concept of functional recovery

Additional file 1: Table S2 summarizes the median scores of the 9 items regarding the concept of functional recovery. The experts did not reach a consensus regarding the existence of a standardized concept of functional recovery in patients with schizophrenia (57.7% of agreement; median score 7.06; 95%CI 6.50–7.50). Nevertheless, the experts in the panel overall agreed on each one of the multiple characteristics frequently included in the definition of functional recovery. For instance, most experts agreed that functional recovery overlaps with other concepts such as quality of life, cognition, and clinical remission. Furthermore, experts identified the influence of symptomatic remission, personal autonomy, professional activity, social relationships, and environmental factors on functional recovery. According to 86.5% of the experts, functional recovery is an achievable goal in patients with schizophrenia.

### Assessment of functional recovery

According to the experts, the assessment of functional recovery is essential in both the clinical research and clinical practice settings (96.2% of consensus). However, there was no consensus on whether the assessment of functional recovery is commonly included among study objectives of clinical trials in schizophrenia (Additional file 1: Table S3). Regarding the source of information for the assessment of functional recovery, there was strong agreement (98.1%) on the suitability of gathering information from three primary sources: patients, their relatives (and/or caregivers), and clinicians. The experts exhibited heterogeneous knowledge of the functional assessment tools currently available for identifying areas subject to improvement, and planning the management of patients with schizophrenia. Most of them stated that they were familiar with the GAF scale, the Personal and Social Performance scale (PSP), and the second version of the World Health Organization Disability Assessment Schedule (WHODAS 2.0). Conversely, the majority of experts were not familiar with the Social and Occupational Functioning Assessment Scale (SOFAS) or the Health of the Nation Outcome Scale (HoNOS). Beyond the scales used, it was suggested that functional recovery is somehow assessed in routine practice, albeit without any standardized procedure.

### Factors influencing functional recovery

The experts overall agreed that functional recovery is influenced by various environmental factors, including stressful life events, substance abuse, socioeconomic conditions, and family dynamics (Additional file 1: Table S4). However, most of them (90%) acknowledged that none of these factors predicts independently the non-achievement of functional recovery. Other environmental factors such as the type of origin (i.e. migrant/local) and residence (i.e. urban/rural) were not considered to influence functional recovery significantly. Both negative and cognitive symptoms were considered to cause significant impact on functional recovery, with no superiority of either of the two symptom groups. Finally, the experts agreed that self-stigma (or internalized stigma) has a greater impact on functional recovery than social stigma. The idea that the negative image associated with psychiatry compared to other medical specialties increases stigma in patients with schizophrenia also reached a consensus in agreement.

### Psychosocial interventions and functional recovery

There was overall agreement that psychosocial interventions are necessary to achieve functional recovery (92.3% of agreement). Among all interventions proposed, family interventions and those aimed at developing social skills and improving employability were considered the most useful for functional recovery (Additional file 1: Table S5). Although the inclusion of cognitive rehabilitation in psychosocial interventions was considered useful, cognitive disorders were not agreed to be the primary target of these interventions.

### Pharmacological treatment and functional recovery

The majority of experts in the panel (79.0%) considered that functional recovery is one of the most important criteria determining the choice of pharmacological treatment (Additional file 1: Table S6). It was also agreed that the various antipsychotic agents have different impacts on functional recovery. Among the potential drawbacks of pharmacological treatment for achieving functional recovery, cognitive impairment reached the highest agreement (100%); 94.3% of the experts agreed that second-generation are more useful than first-generation (or atypical) antipsychotics for achieving functional recovery.

### The perspective of patients and their relatives on functional recovery

The experts agreed that the perspective of clinicians on functional recovery differed significantly from that of patients and their relatives (Additional file 1: Table S7). According to this observation, psychiatrists are more concerned with the clinical aspects of the disease, whereas patients and their relatives are more concerned with subjective aspects of the lifetime project and factors influencing activities of daily living.

## Discussion

Following a two-round Delphi dynamics approach, we found high homogeneity in the opinion of clinicians regarding functional recovery in patients with schizophrenia. Psychiatrists from different areas in Spain achieved consensus in 85% of the concepts addressed regarding various aspects of functional recovery.

Functional recovery is a complex, multidimensional concept to be considered not only by clinicians but also researchers, patients and caregivers, as well mental health policy makers. Although the perspective of the various stakeholders involved in the definition of functional recovery may converge on many aspects, the lack of a common terminology and the pursuit of different goals has led to a wide repertoire of definitions, none of which stands out clearly over the rest [2, 6, 7, 16, 26]. The result of our consensus regarding the concept of functional recovery mirrored this scenario, resulting in a lack of consensus regarding a well-established concept of functional recovery. Nevertheless, the general agreement on specific factors influencing the concept of functional recovery suggests that despite the lack of a standardized definition of recovery, most clinicians share a common archetype of what functional recovery actually is.

The feasibility of achieving functional recovery in patients with schizophrenia has been under discussion since the emergence of interest in this concept [2, 6, 22, 27]. Most experts in our panel (87%) agreed that functional recovery is a realistic goal in the management of patients with schizophrenia. This is in line with the results of recent research on schizophrenia, which showed that psychological well-being and mental health recovery can improve in individuals with first-episode psychosis [28]. The lack of a clear definition and assessment tools prevents from drawing strong conclusions regarding the feasibility of a therapeutic model based on the concept of recovery. However, empirical evidence on various therapeutic interventions suggests that many patients with schizophrenia can achieve goals related to functional recovery such as independent living and competitive employment and education in routine community settings [18–21, 29]. In line with the common perception regarding the definition of functional recovery, M. Farkas proposed four key values commonly reflected in the recovery literature which should be considered in all recovery-oriented services: person orientation, person involvement, self-determination/choice, and growth potential [30].

The lack of a standardized definition is probably a bottleneck for the development of validated tools for the assessment of functional recovery. Other difficulties that may compromise an appropriate assessment of functional recovery include the limitations of some informants to make accurate judgments [31], the limited capacity of some patients for self-assessment [32], and the heterogeneity in their clinical course, which may lead to inconsistencies between the outcome of functioning scales and milestone achievement in some patients (e.g., in some patients, functioning scales may not capture milestone achievements in social, vocational, and residential domains of patients with schizophrenia) [33]. Regarding the source of information for the assessment of functional recovery, there was strong agreement on the suitability of gathering information from three primary sources: patients, their relatives (and/or caregivers), and clinicians. Indeed, some authors have warned of the risk of bias associated with motivation-related negative symptoms (e.g. emotional withdrawal, passive-apathetic social withdrawal) [34]. Furthermore, patient-reported assessments of quality of life and everyday abilities have shown poor correlation with information about lifetime achievements in many patients with schizophrenia [35]. All these limitations are consistent with the lack of consensus on the concept that the patient is the most reliable source of information for functional assessment.

Due to the absence of a single tool for the assessment of functional recovery, clinicians and researchers use different strategies to evaluate it. In an attempt to broaden functional assessment towards a comprehensive model of functional recovery, researchers have combined commonly used scales such as the Global Assessment Functioning (GAF) scale and Global Assessment Scale (GAS) with the Social Functioning Rating Score – which includes both social skills and social roles – and other objective indicators of lifetime achievements [36–38]. The experts exhibited heterogeneous knowledge of the functional assessment tools currently available for identifying areas subject to improvement, and planning the management of patients with schizophrenia. Beyond the scales used, it was suggested that functional recovery is somehow assessed in routine practice, albeit without any standardized procedure. In this regard, treatments based on a recovery model should be consistent with evidence-based treatments [2].

Functional recovery, may be influenced by multiple factors. According to the experts, these factors are a combination of environmental factors, stressful life events, substance abuse, socioeconomic conditions, and family dynamics. Other environmental factors such as the type of origin (i.e. migrant/local) and residence (i.e. urban/rural) were not considered to influence functional recovery significantly. Some authors have observed that patients living in rural areas tend to show better functional outcomes, probably due to greater family and social support as well as simpler vocational roles [39]. However, in our consensus, the experts’ opinion might be strongly influenced by the area where they work. Thus, while some centers provide mental health care to patients from both rural and urban areas, most of them serve one or the other type, whereby the influence of this factor may be unnoticed. Finally, there was 96% agreement that, despite the different perspectives of clinicians and patients (i.e. clinicians tend to focus on the clinical aspects of recovery, whereas patients and their relatives attach importance to the activities of daily living and life project), the attitude of the various stakeholders has an influence on functional recovery.

In line with the results of clinical studies, which suggest that both negative symptoms and cognitive deficits may be primary predictors of impaired social and vocational performance [34, 40, 41], the experts in the panel agreed that both negative and cognitive symptoms cause a significant impact on functional recovery. Also, in agreement with recent recommendations to treat negative symptoms [42], the experts agreed that functional recovery should not be addressed only through symptoms but also considering the cognitive, emotional, and relationship difficulties.

Stigma is another factor with potential influence on functional recovery, and it is generally accepted that it has a major impact on self-esteem and hampers recovery in people with mental illnesses [7, 43]. The experts agreed that the negative image associated with psychiatry compared to other medical specialties increases stigma in patients with schizophrenia and that self-stigma (or internalized stigma) has a greater impact on functional recovery than social stigma. Although the mechanisms of stigma are not clear, social (or public) stigma and self-stigma might work in different ways. In an interview-based study conducted on patients with major depression or schizophrenia, social stigma showed a trend towards underestimating the importance of informal caregivers (e.g. family and friends). Conversely, self-stigma had a negative impact on the perceived importance of seeking help provided by a general practitioner or a psychiatrist [44].

The relevance of psychosocial interventions agreed in this consensus are consistent with the positive results of these interventions reported in randomized clinical trials conducted according to the gold standards of clinical design [45–47]. Although the items regarding the type of therapy with highest effectivity were written in an exclusive way, the experts achieved consensus in the highest effectivity of social skills training, family therapy, cognitive rehabilitation, social cognitive training, and occupational programs. This result indicates that, irrespective of the median score achieved in each therapy, none of them stood out from the rest. Of note, recovery-based interventions are not widespread in clinical practice and some authors have stressed the need to develop more interventions going beyond symptom reduction [48]. Although the inclusion of cognitive rehabilitation in psychosocial interventions was considered useful, cognitive disorders were not agreed to be the primary target of these interventions. The apparent inconsistency regarding the role of cognitive functioning in psychosocial interventions can be explained by the recent evolution of the concept of cognition. Thus, while the construct of cognitive impairment has been traditionally built solely on basic neurocognition, it is now accepted that social cognition differs from basic neurocognition and that it could be the link between neurocognition and functional recovery in psychosocial programming [5, 49].

The positive impact of long-acting antipsychotics on adherence and the closer relationship between patients and the healthcare team associated with the dosing of these agents have been considered helpful for achieving functional recovery [38]. Some authors have questioned the suitability of maintaining long-lasting treatment with antipsychotics [50]. However, the impact of long-lasting antipsychotic treatments on functional recovery is unclear, and other authors have highlighted important limitations of studies investigating early discontinuation of antipsychotic therapy [51].

Although it is not clear whether medication alone can impact directly on functional performance, there is long-time evidence on the synergistic effect of pharmacological and psychosocial treatments, particularly pharmacological treatments with a significant impact on positive symptoms [7, 52–54]. Besides attenuating the symptomatology associated with schizophrenia, pharmacological treatments – particularly atypical antipsychotic agents – cause morphological changes in patients’ brains which could be associated with an improvement in neurochemical functioning [55, 56]. Despite the proven usefulness of some antipsychotic agents in achieving functional recovery [57, 58], the experts identified potential drawbacks of pharmacological treatment for achieving functional recovery: extrapyramidal symptoms, sedation, the worsening of negative symptoms, and cognitive impairment. Of note, most of the adverse events limiting functional recovery are more frequently associated with first-generation than second-generation antipsychotics [59–61]. Combination antipsychotic therapy was also considered to result in poorer functional recovery than monotherapy.

The scope of the results presented herein must be weighed considering some limitations of our work. First, the selection of experts was neither systematic nor randomized. Alternatively, we recruited specialists in the management of schizophrenia from various Spanish regions. Thus, although all experts must account at least 10 years of clinical practice, a selection bias cannot be ruled out. Second, some items expressing mutually incompatible ideas yielded inconsistent results. Items affected by this phenomenon were discussed and eventually not considered for drawing the final conclusions. Finally, the resulting recommendations were not drawn following a consensus process, but as an interpretation of the agreements and disagreements resulting from the Delphi process. Nevertheless, due to the expected heterogeneity on the concept, we deemed it more appropriate to address the conclusions by weighing the scope of each result carefully and addressing the inconsistencies that might arise from the responses of the panel of experts.

## Conclusions

Despite the lack of a standardized definition of functional recovery in schizophrenia, clinicians are aware of this approach, show a trend towards a common construct of this concept, and consider functional recovery in their day-to-day practice, albeit in a non-formal way. In our experience, 57 Spanish psychiatrists reached a consensus on 85% of items addressing various aspects of functional recovery in schizophrenia. Based on the results of this consensus and their consistency with the information available in the literature, the experts of this panel provide a list of recommendations (Table 2).

**Table 2:** List of recommendations when addressing functional recovery of patients with schizophrenia

The concept of functional recovery	
Despite the lack of a unified definition of functional recovery, it is recommended to ponder quality of life, cognition and clinical remission when considering functional recovery in research and routine practice.	
Functional recovery should be considered a goal in the management of patients with schizophrenia.	
Functional recovery should be always included among endpoints of clinical trials assessing patients with schizophrenia.	
Assessment of functional recovery	
Irrespective of the tools used for assessing functional recovery, information for appraising it should be gathered from patients, their relatives (and/or caregivers), and the healthcare team.	
Irrespective of the tools used for assessing functional recovery, the patient’s socio-cultural background should be considered when assessing functional recovery.	
Factors influencing functional recovery	
When seeking for the achievement of functional recovery, the combined influence of stressful life events, substance abuse, socioeconomic conditions, and family relationships, should be considered.	
Although negative symptoms have a great impact on functioning, clinicians should not focus exclusively on symptom remission when considering functional recovery.	
Psychosocial interventions and functional recovery	
Psychosocial interventions are necessary to achieve functional recovery. A combination of various therapies (including social skills training, family therapy, cognitive rehabilitation, social cognitive training, and occupational programs) is likely to be most useful in achieving functional recovery.	
Pharmacological treatment and functional recovery	
Functional recovery should be considered in decision-making on pharmacological treatments.	
The perspective of patients and their relatives on functional recovery	
The attitudes of all stakeholders (i.e., patients, their relatives, and clinicians) influence functional recovery. Hence, when seeking for achieving functional recovery, all these perspectives should be taken into account.	

## Additional file

Additional file 1: Results of the Delphi consensus. Score (median and interquartile range) of each item of the questionnaire. (PDF 397 kb)

## Abbreviations

GAF: Global Assessment Functioning
GAS: Global Assessment Scale
HoNOS: Health of the Nation Outcome Scale
PSP: Personal and Social Performance
SOFAS: Social and Occupational Functioning Assessment Scale
WHODAS: World Health Organization Disability Assessment Schedule
