# Supplementary material for: Functional recovery in patients with schizophrenia: recommendations from a panel of experts
Source: BMC Psychiatry. 2018 Jun 5;18:176. doi: 10.1186/s12888-018-1755-2 (PMC5989342; doi:10.1186/s12888-018-1755-2)

Functional recovery in patients with schizophrenia. Recommendations from a panel of experts

Additional file 1 – Results of the Delphi consensus

Table S1 – Consensus criteria

**Consensus**
≥ 1/3 of experts score EITHER<br>7 – 9 (agree)<br>OR<br>1 – 3 (disagree)
**Non-consensus**
Interquartile range > 4<br>OR<br>≥ 2/3 of experts score 1 – 3<br>AND<br>≥ 2/3 of experts score 7 – 9
**Indeterminate**
Criteria for either consensus or non-consensus is not met

Median
Interquartile range

Consensus in agreement
Consensus in disagreement
No consensus

Table S2 – The concept of functional recovery

01. Functional recovery is a well-established concept.

02. The concept of functional recovery overlaps with other concepts such as quality of life, cognition, recovery or clinical remission.

03. Functional recovery is influenced by symptomatic remission.

04. Functional recovery is influenced by personal autonomy.

05. Functional recovery is influenced by occupational activity.

06. Functional recovery is influenced by social relationships.

07. Functional recovery is influenced by subjective factors.

08. Functional recovery is influenced by environmental factors

09. Functional recovery is achievable in patients with schizophrenia.

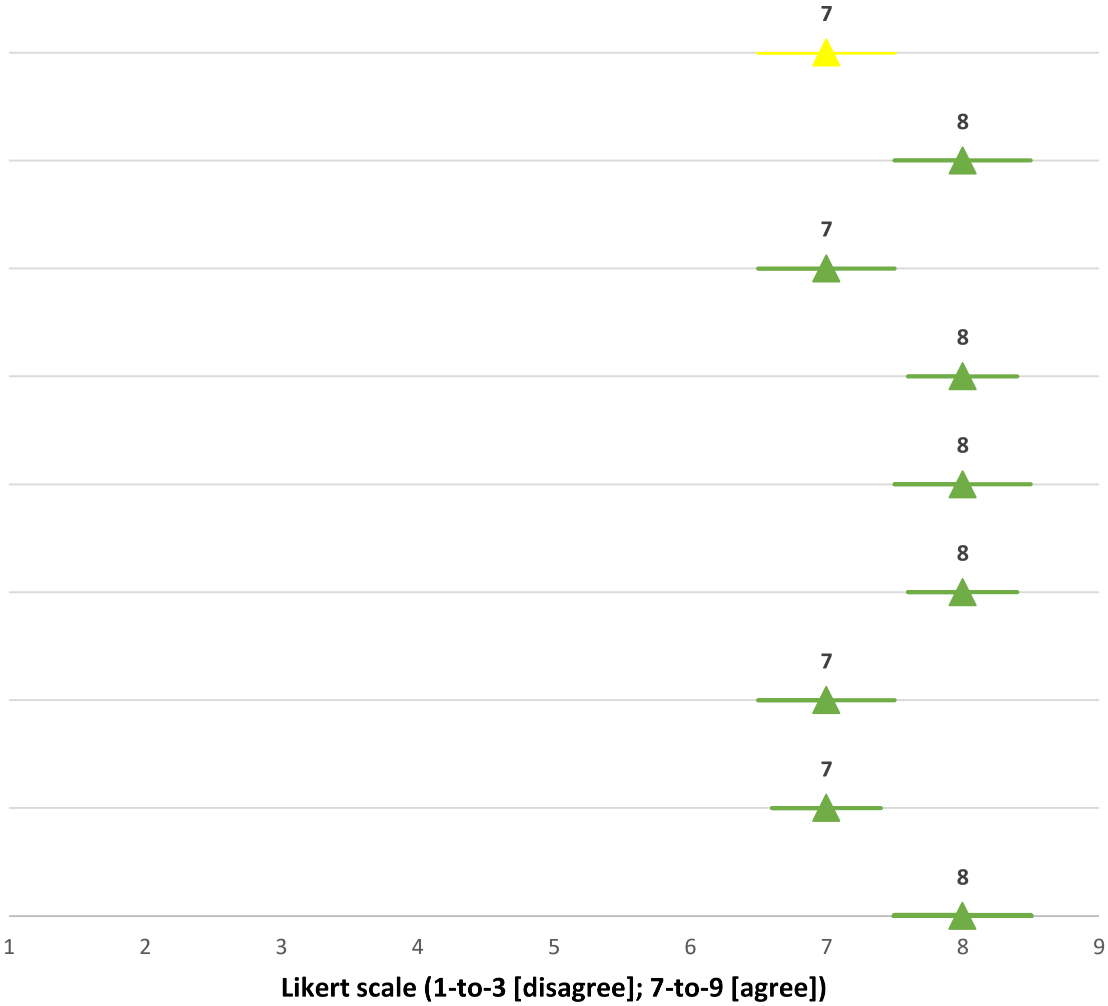

Table S3 – Assessment of functional recovery

10. Functioning must be always considered in the assessment of patients with schizophrenia.
11. A functional assessment is commonly included among the objectives of clinical trials in schizophrenia.
12. A functional assessment should be included among the objectives of clinical trials in schizophrenia.
13. Patients with schizophrenia and poor insight tend to overestimate their functional performance.
14. Functional assessment is challenging due to the overlapping with other concepts such as quality of life and clinical remission.
15. The patient is the most reliable source of information for functional assessment.
16. The patient's relatives (or caregivers) are the most reliable source of information for functional assessment.
17. The healthcare team is the most reliable source of information for functional assessment.
18. In the clinical practice setting, gathering information from all sources (i.e. the patient, his/her caregiver, and the healthcare team) is the most reliable and useful option.
19. In clinical research settings, retrieving information from all three sources (i.e. the patient, his/her caregiver, and the healthcare team) is the most reliable and useful option.
20. A functional assessment should consider the patient's socio-cultural background.
21. A proper functional assessment is not affordable in institutionalized patients.
22. The use of assessment tools for the functional evaluation of patients with schizophrenia is uncommon.
23. Many assessment tools are available for the functional evaluation of patients with schizophrenia.
24. There is no standardized tool for the functional assessment of patients with schizophrenia.
25. I am familiar with the use of the Global Assessment Functioning (GAF) scale regarding the functional assessment of patients with schizophrenia.
26. I am familiar with the use of the Social and Occupational Functioning Assessment Scale (SOFAS) regarding the functional assessment of patients with schizophrenia.
27. I am familiar with the use of the Personal and Social Performance (PSP) scale regarding the functional assessment of patients with schizophrenia.
28. I am familiar with the use of the Schizophrenia Outcomes Functioning Interview (SOFI) regarding the functional assessment of patients with schizophrenia.
29. I am familiar with the use of the Health of the Nation Outcome Scale (HoNOS) regarding the functional assessment of patients with schizophrenia.
30. I am familiar with the use of the second version of the World Health Organization Disability Assessment Schedule (WHODAS 2.0) regarding the functional assessment of patients with schizophrenia.
31. I am familiar with the use of the UCSD Performance-based Skills Assessment (UPSA) regarding the functional assessment of patients with schizophrenia.
32. Spanish clinicians are familiar with functional assessments based on self-administered scales.

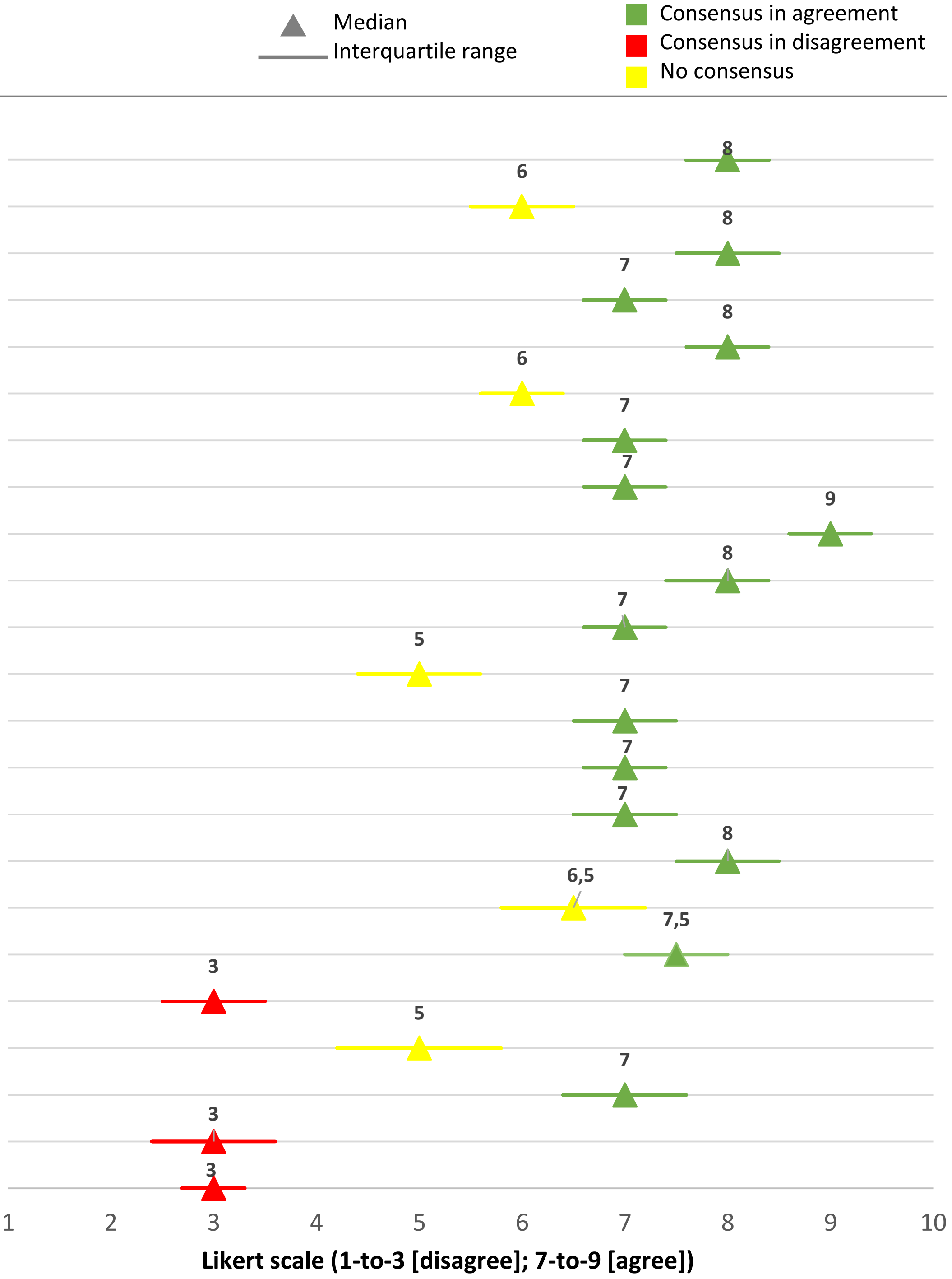

Table S4 – Factors influencing functional recovery

33. In patients with schizophrenia, functional recovery should be addressed through a comprehensive approach that includes cognitive, emotional, and relationship difficulties, rather than focus on the symptoms.

34. Negative symptoms have the greatest impact on the functioning of patients with schizophrenia.

35. In patients with schizophrenia, cognitive symptoms have the most significant impact on functioning.

36. In patients with schizophrenia, impaired social cognition has a greater impact on functioning than overall neuropsychological impairment.

37. Stressful life events are the environmental factor with the greatest impact on functioning in patients with schizophrenia.

38. Substance abuse is the environmental factor withe the greatest impact on functioning in patients with schizophrenia.

39. Socio-economic conditions are the environmental factor with the greatest impact on functioning in patients with schizophrenia.

40. Family dynamics are the environmental factor with the greatest impact on functioning in patients with schizophrenia.

41. Living in an urban area is the environmental factor with the greatest impact on functioning in patients with schizophrenia.

42. Being an immigrant is the environmental factor with the greatest impact on functioning in patients with schizophrenia.

43. None of the above-mentioned environmental factors can independently predict poor functional recovery in patients with schizophrenia.

44. Self-stigma (or internalized stigma) has a greater impact on functioning than social stigma.

45. Public campaigns aimed at reducing stigma in patients with schizophrenia are effective.

46. The poor image of psychiatry compared to other medical specialties increases stigma in patients with schizophrenia.

47. Functional recovery is possible without an awareness of the disease.

48. Adherence to both antipsychotic treatments and psychosocial interventions is necessary for functional recovery.

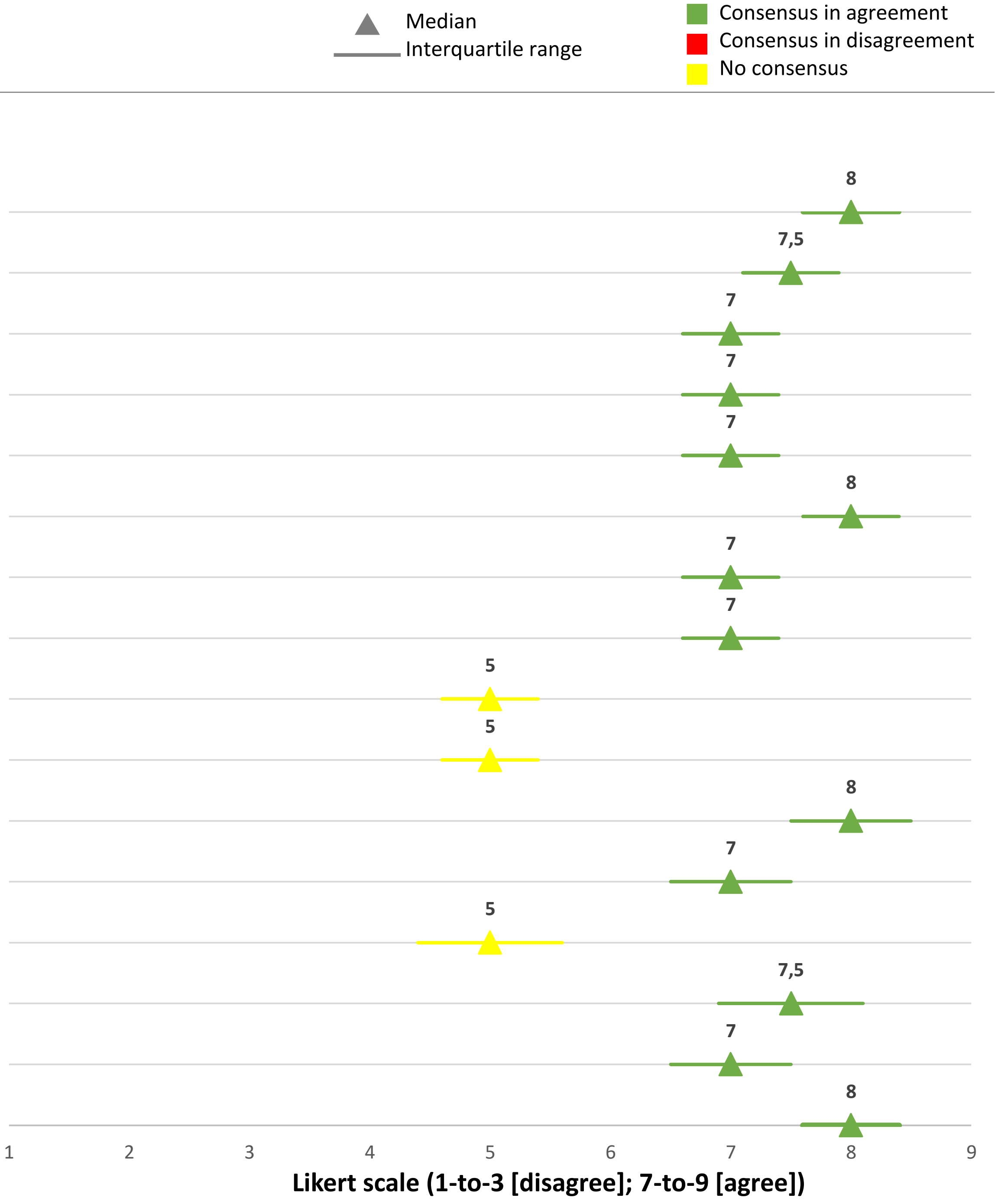

Table S5 – Psychosocial interventions and functional recovery

Median
Interquartile range

Consensus in agreement
Consensus in disagreement
No consensus

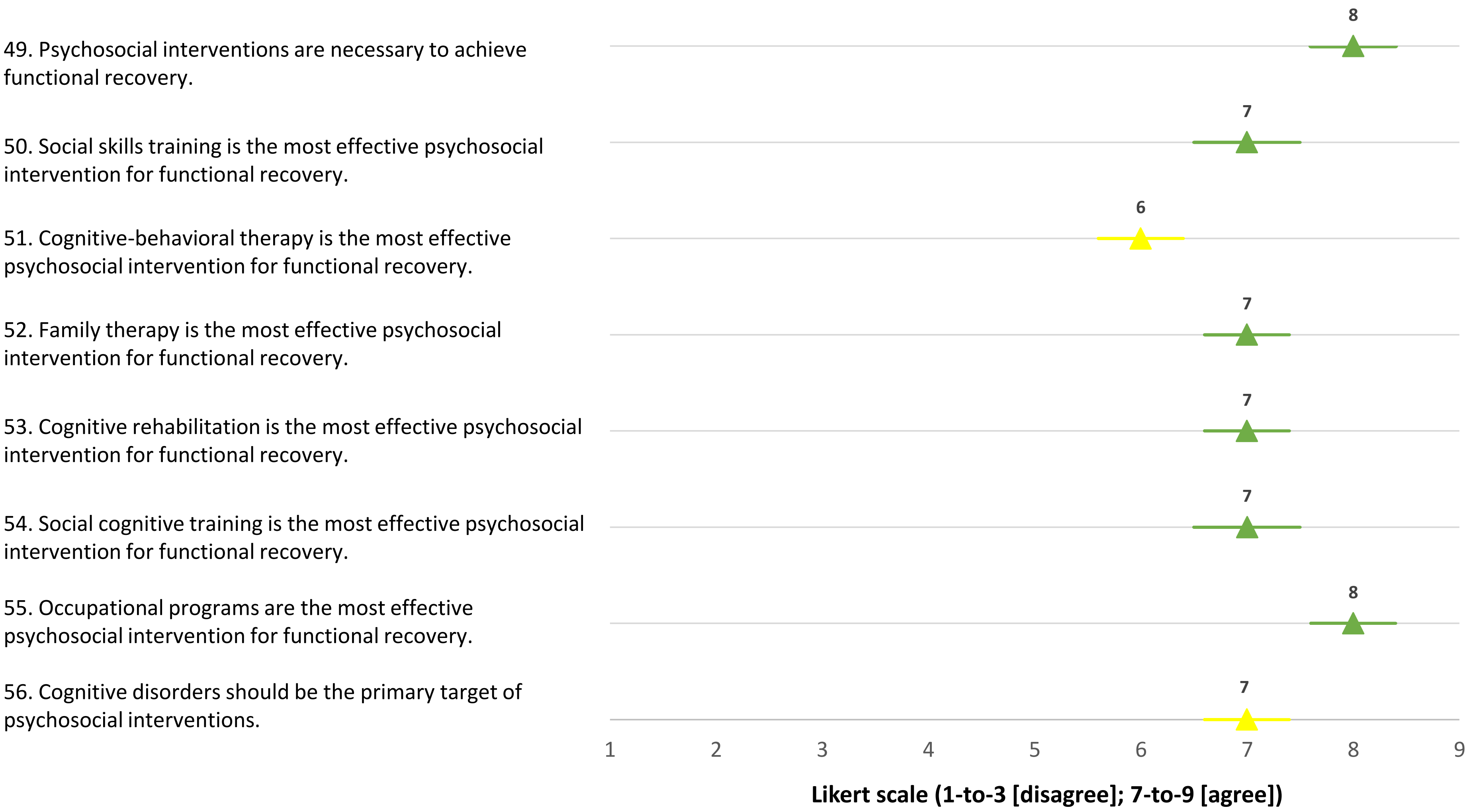

Table S6 – Pharmacological treatment and functional recovery

57. Functional recovery is one of the most important criteria for choosing a pharmacological treatment.

58. There are differences between the various antipsychotics regarding their effect on functional recovery.

59. Compared to conventional antipsychotics, treatment with atypical antipsychotics is associated with greater rates of functional recovery.

60. Functional recovery is hampered by adverse events such as extrapyramidal symptoms.

61. Functional recovery is hampered by adverse events such as sedation.

62. Functional recovery is hampered by adverse events such as the onset/worsening of negative symptoms.

63. Functional recovery is hampered by adverse events such as cognitive impairment.

64. Compared with monotherapy, combined antipsychotic treatment is associated with poorer functional recovery.

65. The efficacy of antipsychotic agents in the remission of positive symptoms is an essential quality for their activity on functional recovery.

66. The efficacy of antipsychotic agents in the remission of negative symptoms is an essential quality for their activity on functional recovery.

67. Relapse prevention is an essential quality of antipsychotic agents regarding their activity on functional recovery.

68. The primary role of antipsychotic agents is to facilitate and complement psychosocial rehabilitation interventions.

69. Long-acting injectable antipsychotic agents benefit functional recovery by promoting interaction between patients and mental care teams.

70. Long-acting injectable antipsychotic agents benefit functional recovery by improving treatment adherence.

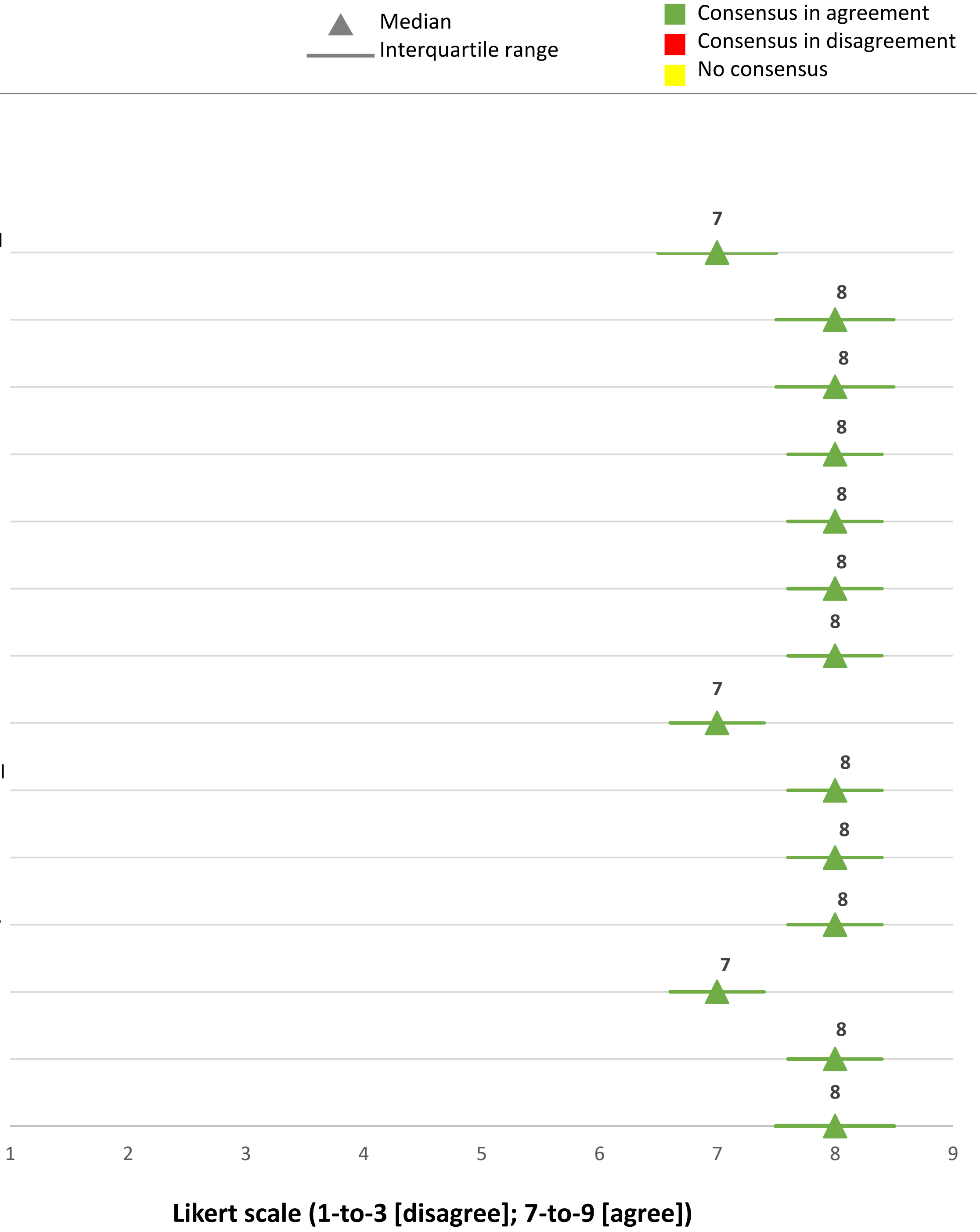

Table S7 – The perspective of patients and their relatives on functional recovery

Median
Interquartile range

Consensus in agreement
Consensus in disagreement
No consensus

71. The perspective of clinicians on functional recovery differs significantly from that of patients and their relatives.

72. Regarding functional recovery, psychiatrists are more concerned with the clinical aspects of the disease.

73. Regarding functional recovery, patients' relatives are more concerned with factors related to the activities of daily living.

74. Regarding functional recovery, patients are more concerned with subjective aspects of their lifetime project.

75. The attitude of the various stakeholders (clinicians, patients, relatives, and other professionals involved) influences functional recovery.

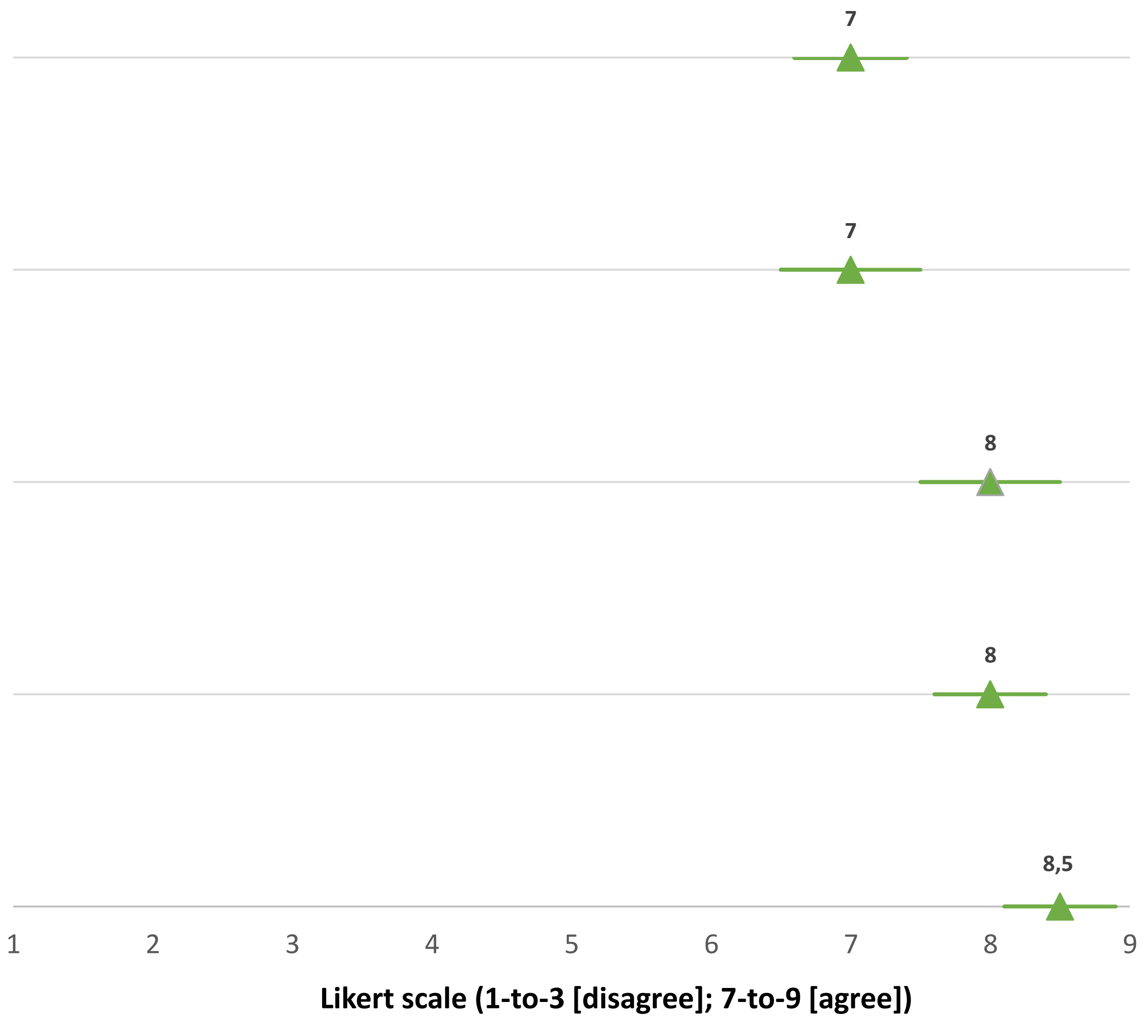

Supplement: Supplementary file 1 — Results of the Delphi consensus. Score (median and interquartile range) of each item of the questionnaire. (PDF 397 kb) [file 12888_2018_1755_MOESM1_ESM.pdf]
